# Myricetin Abrogates Atrazine‐Mediated Inflammation, Cognitive Decline, and Alzheimer's Disease‐related pathology in a Rat model of Parkinson's Disease Dementia

**DOI:** 10.1002/alz70855_096935

**Published:** 2025-12-23

**Authors:** Cynthia Nwamaka Ikeji, Ebenezer Olatunde Farombi

**Affiliations:** ^1^ University of Ibadan, Ibadan, Oyo, Nigeria

## Abstract

**Background:**

Parkinson's disease and its related dementia (PDD), a non‐motor complication associated with late onset is fast affecting Africa's older populace. Meanwhile, the herbicide atrazine, has been linked to the pathogenesis of Parkinson's Disease. Myricetin which is a flavonoid found in vegetables and fruits is reputed for its myriad ranges of biological activities, neuro‐protection included. Therefore, this study aimed at investigating the neuro‐protective role of myricetin on atrazine‐mediated inflammation, memory deficit, and neurodegeneration in a rat model of Parkinson's Disease Dementia.

**Method:**

Thirty‐two adult male Wistar rats (250 g‐ 300 g) were sorted into four groups of eight animals each and orally treated with atrazine (PD group), myricetin, and a combination of both at 50 and 20 mg/kg. b.w. respectively for 65 days. Post treatment, behavioral tests on cognition was conducted using Morris Water Maze before euthanasia, and the assessment of nigral alpha‐synuclein (SNCA), striatal tyrosine hydroxylase, and hippocampal amyloid beta protein expression, as well as hippocampal inflammatory markers (nitric oxide (NO), inducible nitric oxide synthase (INOS), ionized calcium‐binding adaptor molecule (IBA‐1)) were assessed using both chromogenic immunohistochemistry and spectrophotometry techniques, incorporating bright‐field microscopy.

**Result:**

Animals exposed to 50 mg/kg of atrazine showed a significant upregulation of amyloid beta 42 (Aβ‐42), a molecular marker associated with AD pathogenesis. Additionally, atrazine impaired both memory retention and consolidation, through the inability of animals to locate the target quadrant and decrease in time spent in target quadrant, suggesting cognitive decline. To assess potential therapeutic strategies, we co‐treated animals with 20 mg/kg of myricetin, a flavonoid known for its neuroprotective properties. Myricetin treatment successfully reversed the deficits induced by atrazine, significantly reducing the levels of Aβ42, NO, INOS, IBA‐1, SNCA, while improving memory retention and consolidation times as indicated increase by the number of platform zone entries and time spent in MWM platform quadrant in the co‐treatment group of myricetin and atrazine compared with the atrazine/PD group.

**Conclusion:**

These findings demonstrate that myricetin has the potential to counteract the neurotoxic effects of atrazine, providing a promising avenue for developing therapeutic interventions for environmental toxin‐induced cognitive impairments associated with Parkinson's Disease.

